# Insecure adult attachment and reflective functioning as mechanisms of the relationship between traumatic life events and suicidal ideation: A path analysis

**DOI:** 10.3389/fpsyg.2022.985148

**Published:** 2022-09-30

**Authors:** Alessandro Musetti, Luca Pingani, Andrea Zagaria, Daniele Uberti, Salvatore Meli, Vittorio Lenzo, Alessio Gori, Christian Franceschini, Gian Maria Galeazzi

**Affiliations:** ^1^Department of Humanities, Social Sciences and Cultural Industries, University of Parma, Parma, Italy; ^2^Department of Biomedical, Metabolic and Neural Sciences, University of Modena and Reggio Emilia, Modena, Italy; ^3^Dipartimento ad attività integrata Salute Mentale e Dipendenze Patologiche, Azienda USL-IRCCS di Reggio Emilia, Reggio Emilia, Italy; ^4^Department of Psychology, Sapienza University of Rome, Roma, Italy; ^5^Dipartimento di Scienze della Società e della Formazione d’Area Mediterranea, Università per Stranieri Dante Alighieri, Reggio Calabria, Italy; ^6^Department of Health Sciences, School of Psychology, University of Florence, Firenze, Italy; ^7^Department of Medicine and Surgery, University of Parma, Parma, Italy

**Keywords:** suicidal ideation, suicide risk, traumatic experiences, attachment, reflective functioning, mentalization

## Abstract

The relationship between traumatic life events and increased suicide risk has been well reported in literature. However, the complex nature of suicidality phenomena still hinders our ability to comprehend the mediation mechanism underlying this association. In this study, we examined the mediating role of adult attachment and reflective functioning in the relationship between traumatic life events and suicidal ideation. Nine hundred and fifty Italian adults completed an online survey evaluating traumatic life events, adult attachment, reflective functioning and suicidal ideation. The path analysis showed that the positive relationship between traumatic life events and suicidal ideation was partially mediated by attachment anxiety and reflective functioning. From a clinical point of view, these results support the relevance of evaluating and improving patients’ ability to mentalize as a part of psychotherapeutic intervention aimed at reducing suicidality in people with a history of traumatic experiences and attachment anxiety.

## Introduction

Suicide (i.e., the act of voluntarily and intentionally causing oneself death) is a major global public health issue. According to World Health Organization (2021) statistics, nearly 703,000 people committed suicide in 2019, making suicide one of the leading causes of death worldwide with 1.3% of global deaths. Suicide is a complex phenomenon intertwined with biological, psychological, social, and environmental factors (Turecki et al., 2019). As for gender, although suicidal ideation and attempts are more frequent among females, men are more likely to complete suicide than women—a phenomenon referred to as “the gender paradox” in suicide (Canetto and Sakinofsky, 1998; Weissman et al., 1999; Bernal et al., 2007). Relatedly, a systematic review and meta-analysis on contact with mental health services prior to suicide (Walby et al., 2018) found higher rate of admission to psychiatric care in the year before death among females compared to males. This could be related to traditional gender norms (e.g., men’s desire to appear strong) and male mental health-related stigma (e.g., mental illness as a form of weakness) as deterrents to seek help from mental health services (Clement et al., 2015). More in general, gender differences in suicidality may be in part explained by behavioral factors (e.g., males’ preference for highly lethal suicide methods; Varnik et al., 2008; Mergl et al., 2015) and differences in the experience and expression of emotional difficulties, such as the higher rates of externalizing and internalizing problems in males and females, respectively (Mergl et al., 2015; Leban, 2021).

Suicidality includes a spectrum of diverse phenomena ranging from death thoughts and wishes (i.e., suicidal ideation) to suicide behaviors (i.e., suicide attempts and completions). Specifically, the term suicidal ideation refers to cognitions that can vary from transient death wishes to detailed plan for killing oneself (Liu and Miller, 2014). Suicidal ideation is considerably more frequent than suicidal behaviors (Nock et al., 2008). Although suicidal ideation is a clear risk factor for suicide behaviors, the majority of ideators do not attempt or complete suicide (Glenn and Nock, 2014; Klonsky et al., 2016). Variability of suicide phenomena and their extensive comorbidity with other mental disorders makes an exact etiology difficult to determine, and thus further research is needed.

### Traumatic life events and suicidal ideation

Research has identified a variety of risk and protective factors for suicidal ideation, including sociodemographic (e.g., younger age, not being married/co-habiting), socio-economic (e.g., unemployment, economic hardship), and clinical factors (e.g., mood disorders, impulse-control disorders, and substance disorders) (Nock et al., 2008; Hintikka et al., 2009). A large body of literature has been accumulated showing that traumatic life events (TLE) are distal and proximal risk factors for suicidal ideation (Krysinska and Lester, 2010; Sorsdahl et al., 2011; Liu and Miller, 2014; Beristianos et al., 2016; Turecki and Brent, 2016; Ásgeirsdóttir et al., 2018).

In the fifth edition of the Diagnostic and Statistical Manual of Mental Disorders (DSM-5), a traumatic event is defined as both direct and indirect “exposure to an actual or threatened death, serious injury or sexual violence” (American Psychiatric Association, 2013). Although this definition is widely used in clinical practice and research, some scholars (Capraro, 2016; Schimmenti, 2018) have raised concerns about the DSM-5’s emphasis on the objective characteristics of adverse exposures. In this respect, available evidence shows relevant individual differences in response to potential traumatic events (Emery et al., 1991). For example, the individual’s response to an adverse event can vary depending on their previous traumatic experiences and the amount of social support received (Fink and Galea, 2015). Moreover, accumulated evidences show that different types of TLE often co-occur and interact increasing negative effects on mental health (Cloitre et al., 2009; Debowska et al., 2017; Bifulco and Schimmenti, 2019).

In an attempt to overcome these limitations, Schimmenti (2018) proposed the “trauma factor” model. Rooted in the psychoanalytic tradition (Khan, 1963), this model introduces the concept of global trauma which refers to the cumulative effect of past and current traumatic experiences. In this vein, psychological trauma is intended as a complex factor which includes, but is not limited to, specific adverse experiences occurring in the individual’s life. Furthermore, Schimmenti (2018) showed that the relationship between TLE and psychopathology is often indirect, and includes a number of mediators, as, for example, attachment. Exploring such mediators could be, therefore, a reasonable strategy for improving knowledge and interventions aiming at reducing mental health problems (suicidal risk included).

### Mediating role of adult attachment

Attachment theory (Bowlby, 1969, 1973, 1980, 1988) is a sound framework for understanding how early parent–child relationships set the stage for later psychological functioning on both the individual and interpersonal levels. Attachment was originally conceptualized by Bowlby as a relatively stable relational bond between infants and their primary birth attachment figures which provides a secure base for exploration of the environment. Within a vulnerability-stress perspective (Davila et al., 2005), the lack of security in childhood attachment relationships could result in severe interpersonal difficulties in forming and maintaining meaningful relationship with others, hindering the propensity to ask for support in times of emotional distress. In support, empirical research has linked developmental traumas (such as failures of care and abusive experiences in attachment relationships) to an increased vulnerability to psychopathology (Davila et al., 2005; Midolo et al., 2020; Musetti et al., 2022).

Different from child attachment, which is observed in infant-caregiver relationships, adult attachment refers to the individual’s view of the self and others in intimate relationships (e.g., romantic partners, close friends) during adult life (Ravitz et al., 2010). Bartholomew (Bartholomew, 1990; Bartholomew and Horowitz, 1991) identified four categories of adult attachment, consisting of (a) secure attachment which reflects a positive view of the self and a positive view of others, (b) preoccupied attachment which reflects a negative view of the self and a positive view of others, (c) fearful attachment which reflects a negative view of the self and a negative view of others and (d) dismissing attachment which reflects a positive view of the self and a negative view of others. These categories (i.e., attachment styles) can be placed in four quadrants, formed by two orthogonal underlying dimensions, namely attachment anxiety and avoidance. Attachment anxiety is characterized by a negative view of the self, fear of rejection and an overwhelming desire for closeness and intimacy. Attachment avoidance entails appearance of confident self-reliance, mistrust of others and discomfort with closeness. Specifically, people with high attachment anxiety are more inclined to detect threats in the world, to exaggerate the potential negative consequences of their actions, and to increase mental polarization on threat-related concerns through hyperactivating strategies (e.g., rumination) that produce a self-amplifying cycle of distress. On the other hand, people with attachment avoidance tend to adopt deactivating strategies (e.g., maintaining of self-reliance and distance) to avoid frustration and further distress related to the unavailability of attachment figures (Mikulincer et al., 2003).

Although adult attachment is thought to reflect relatively stable working models of intimate bonds, it is potentially modifiable by disconfirming experiences of life events (Bowlby, 1988; Hesse et al., 1999). A 6-year longitudinal study by Zhang and Labouvie (2004) found that adult attachment is characterized more by fluidity than by stability. Also, fluctuation in attachment security-insecurity showed concurrent covariations with coping strategies (i.e., constructive versus defensive) and emotional well-being. On the basis of these findings, the authors concluded that changes in adult attachment are probably associated with a profound readjustment of ways of experiencing intimate relationships and interacting with others. In the same vein, another longitudinal study on over 4,000 adults (Fraley et al., 2021), showed that a variety of life events may lead to transient or enduring changes in attachment. Among these life events, TLE may amplify negative models of self and/or others and result in attachment insecurities (Muller et al., 2000; Cozzarelli et al., 2003).

Adam (1994) developed a psychological model of suicide that is rooted in attachment theory. According to this model, individuals with attachment insecurities tend to develop low self-esteem, maladaptive coping strategies, and relationship difficulties. When these trait vulnerabilities are coupled with current experiences of adversity (e.g., loss, bereavement, rejection), an attachment crisis may be triggered. From this standpoint, Adam has conceptualized suicide as an extreme attachment behavior aimed at communicating discomfort and anger toward an unresponsive or unavailable attachment figure. Consistent with this theoretical framework, previous studies found positive associations between insecure attachment and increased risk of suicide, as well as a negative association between secure attachment and suicidal ideation and behaviors (Miniati et al., 2017; Green et al., 2020; Zortea et al., 2021). Moreover, adult attachment was found to mediate the relationship between childhood traumatic experiences and suicidality (Ihme et al., 2022; Stagaki et al., 2022). However, less is known about the relationship between TLE, adult attachment and suicidal ideation.

### Multiple mediating role of adult attachment and reflective functioning

Mentalization, operationalized as reflective functioning (RF), represents a novel and emerging research field in the contest of suicide risk (Green et al., 2021; Stagaki et al., 2022). RF refers to the ability to understand one’s own behavior and the behavior of others in terms of mental states (e.g., feelings, desires, and attitudes). More specifically, hypomentalizing reflects an inability to consider or think about mental states, whereas hypermentalizing reflects the tendency to be overly certain about mental states of self or others (Katznelson, 2014).

Attachment and mentalization are deeply intertwined but not identical constructs (Fonagy and Bateman, 2016). Mentalization has its roots in attachment relationships, so that disruptions of early attachment, such as childhood maltreatment, can compromise the development of an adequate RF (Fonagy and Luyten, 2009). However, it is well known that RF is a highly interactive process. Indeed, although it develops in the context of child-relationship interactions, RF continues to be influenced through interactions with significant others (Luyten et al., 2020). Moreover, RF includes both trait and state features, so that stability in RF may coexist with fluctuations over time and across relationship contexts (Luyten et al., 2020). Previous studies (e.g., Borghesi et al., 2022) found that TLE are associated with RF failures. Indeed, under increased arousal or stress the capacity to use more conscious and reflective strategies (controlled mentalization) can switch towards a more impulsive and non-conscious mental processes (automatic mentalizing) that are activated in those situations which require an immediate response to threats (Luyten et al., 2020).

The relationship between TLE, attachment, and RF has been extensively investigated (Fonagy and Bateman, 2016). Individuals with secure attachment are more prone to use constructive strategies to deal with and regulate negative emotional states linked to traumatic memories (Vrtička and Vuilleumier, 2012). Conversely, individuals with TLE and insecure attachment are more likely to develop impairments in RF, such as epistemic mistrust (i.e., a pervasive mistrust in the information conveyed from others; Bo et al., 2017).

In some studies, RF has been studied as a mediator between adult attachment and psychopathology (Santoro et al., 2021). For instance, Beeney et al. (2015) found that RF mediates the relationship between adult attachment insecurity and antisocial traits. Other research has revealed the mediating role of RF between adult attachment and adaptive psychological features (Nazzaro et al., 2017).

Difficulties in utilizing mental state information to understand oneself and others have been identified in people with suicidal ideation (Bateman and Fonagy, 2012; Vrtička et al., 2012; Long et al., 2020; Luyten et al., 2020; Rohani and Esmaeili, 2020; Levi-Belz and Lev-Ari, 2022). A recent study by Green et al. (2021), has investigated for the first time the mediating role of RF in the relationship between adult attachment and suicidal ideation in a sample of current suicide ideators. Although results did not support the hypothesized mediation model, these preliminary findings deserve to be reconsidered in further studies before being considered conclusive. First, it should be noted that significant positive associations were observed between attachment avoidance and suicidal ideation as well as between attachment anxiety and failures in RF. Second, non-significant associations should be interpreted with caution due to the small sample size, consisting of 64 participants who completed entirely the administered self-report measures. In addition, as highlighted by the authors, the narrow focus on current suicidal ideation do not allow to generalize the results to a broader population (e.g., people with a life-time history of suicidal ideation). These shortcomings call for further research in this area.

### Study purpose

Individuals with a history of TLE are more at risk for suicidal ideation, however, the underlying psychological mechanisms continue to be debated in the literature. To the best of our knowledge, no study has yet investigated the relationship between TLE and suicidal ideation while taking adult attachment and RF into account. Thus, to overcome this gap, we have undertaken the present study. We hypothesized that the severity of suicidal ideation would be positively associated with the severity of TLE (H1) and that this relationship would be partially and positively mediated by insecure adult attachment (i.e., attachment anxiety and avoidance) (H2) and, in sequence, partially and negatively mediated by RF (H3).

## Materials and methods

### Procedure

Participants were recruited through an online snowball convenience sampling, advertising the invitation to participate in the study through announcements on social network platforms published on December 18, 2020 which remained available until February 1, 2021. The online survey was implemented using Google Form. The inclusion criteria were to be at least 18 years old and to be able to understand the Italian language. All participants were informed of the nature of the research and gave their online written consent. After reading the instruction, participants were asked to complete a socio-demographic form (age, gender, educational level, marital status, employment status) and psychometric questionnaires. All the survey responses were mandatory to avoid missing values.

The sample size was determined by using two criteria: (1) the “n: q criterion” (Raykov and Marcoulides, 2006)—number of participants (n) to the number of (free) model parameters to be estimated (q). At least 10 subjects per parameter were guaranteed; (2) to perform a multivariate logistic regression a minimum sample size of 500 respondents was settled following previous recommendation (Bujang et al., 2018).

All procedures contributing to this work comply with the ethical standards of the relevant national and institutional committees on human experimentation and with the Helsinki Declaration of 1975, as revised in 2008.

### Measures

#### Sociodemographic information

Participants completed a form collecting sociodemographic data concerning gender, age, educational level, marital status, and working status.

#### History of suicidal ideation

The Columbia-Suicide Severity Rating Scale (C-SSRS) is a 20-question tool that evaluates suicidal ideation and behavior. For the present study, the C-SSRS was used to assess quantitatively the history of suicidal ideation as a dichotomous outcome calculated as a “yes” answer to any of the five questions (items 1–5 on the C-SSRS, e.g., “Have you thought about being dead or what it would be like to be dead?”) implies the presence of a history of suicidal ideation while no positive response implies its absence. No reverse items are included (Nilsson et al., 2013). The questionnaire demonstrated good convergent and divergent validity with other multi-informant suicidal ideation and behavior scales and had high sensitivity and specificity for suicidality (Posner et al., 2011). The Italian version of the C-SSRS (Stefa-Missagli et al., 2020) has shown good psychometric properties.

#### Reflective functioning

The Reflective Functioning Questionnaire (RFQ) (Fonagy et al., 2016; Italian version by Morandotti et al., 2018) was used to evaluate the participants’ RF. Participants rated the items on a 7-point Likert scale (ranging from “1 – completely disagree” to “7 – completely agree”). RFQ has two subscales, each containing 6 items, which assess certainty (RFQc; e.g., “When I get angry I say things that I later regret”; reverse-scored) and uncertainty (RFQu; e.g., “If I feel unsecure I can behave in ways that put others’ backs up”) about mental states of oneself and others, respectively. Of the 6 items on each subscale (RFQc: 1, 2, 3, 4, 5, 6; RFQu: 2, 4, 5, 6, 7, 8) two are unique and four are shared (2, 4, 5, 6) across the two subscales. All the items of RFQc subscale were rescored (3, 2, 1, 0, 0, 0, 0 with 3 = disagree strongly) such that higher scores indicated higher certainty about mental states. All the items of RFQu subscale except one (i.e., item 7; reverse-scored) were rescored (0, 0, 0, 0, 1, 2, 3; 3 = agree strongly). High scores on RFQu reflect higher uncertainty about mental states (Cucchi et al., 2018). Previous studies demonstrated that internal consistency and test–retest reliability of the subscales were satisfactory to excellent (Fonagy et al., 2016; Müller et al., 2022). The Italian version (Morandotti et al., 2018) confirmed the *a priori* factor structure showing two subscales that measure certainty and uncertainty about mental states, with satisfactory reliability and construct validity.

#### Adult attachment

The Relationship Questionnaire (RQ) (Bartholomew and Horowitz, 1991; Italian version by Carli, 1995) is a 4-item scale designed to measure four adult attachment using a 7-point Likert scale (1 = disagree strongly; 4 = neutral mixed; 7 = agree strongly) as follows: (a) secure attachment style (“It is easy for me to become emotionally close to others. I am comfortable depending on them and having them depend on me. I do not worry about being alone or having others not accept me”); (b) fearful attachment style (“I am uncomfortable getting close to others. I want emotionally close relationships, but I find it difficult to trust others completely, or to depend on them. I worry that I will be hurt if I allow myself to become too close to others”); (c) preoccupied attachment style (“I want to be completely emotionally intimate with others, but I often find that others are reluctant to get as close as I would like. I am uncomfortable being without close relationships, but I sometimes worry that others do not value me as much as I value them”); (d) dismissing attachment style (“I am comfortable without close emotional relationships. It is very important to me to feel independent and self-sufficient, and I prefer not to depend on others or have others depend on me). Based on previous studies (Griffin and Bartholomew, 1994; Wongpakaran et al., 2021) the following dimensions were used for this study: attachment anxiety [(B + C)–(A + D)] and attachment avoidance [(B + D)–(A + C)]. Higher scores indicate higher levels of the assessed attachment styles or dimensions. The RQ is a valid and well-validated measure with good test–retest reliability (Ligiéro and Gelso, 2002) and good psychometric properties in the general population in different cultural contexts and languages. The Italian version validated by Carli showed good psychometric properties.

#### Traumatic life events

The Traumatic Experience Checklist (TEC) (Nijenhuis et al., 2002; Italian version by Schimmenti, 2018) is a self-report measure to assess the frequency of TLE. The questionnaire consists of 29 items (e.g., “Having to look after your parents and/or brothers and sisters when you were a child”). The possible answers can be “Yes” (Score = 1) or “No” (Score = 0). The TEC has demonstrated adequate reliability and validity in Italian and international studies (Nijenhuis et al., 2002; Craparo et al., 2013; Schimmenti et al., 2017). For our study, we used the cumulative score as an index of total trauma exposure: the higher the score, the higher the number of TLE reported.

### Analytic plan

Data were analyzed through IBM SPSS 22 (IBM Corporation, Armonk NY; USA) and Mplus 8.6 (Muthén and Muthén, 1998–2017). Continuous variables were described using mean and standard deviation, whereas categorical variables (ordinal and nominal) using absolute and percentage frequencies. Normality assumptions were assessed by calculating skewness and kurtosis values (e.g., Hair et al., 2010). Preliminarily to model testing, mean differences across history of suicidal ideation categories (i.e., presence of suicidal ideation vs. absence of suicidal ideation) on the continuous variables under investigation were assessed by carrying out a series of independent samples *t*-tests, while relationships between suicidal ideation subgroups and the categorical variables under study were assessed by implementing Chi-square tests of independence. Also, Pearson’s correlation coefficients were calculated between the suicidal ideation summed score and the continuous variables under investigation. Subsequently, a multiple logistic regression was performed by setting the dichotomous variable of a history of suicidal ideation as the model outcome (i.e., presence vs. absence of suicidal ideation), evaluating the predictive role of the RFQc, RFQu, attachment anxiety, and attachment avoidance dimensions, as well as of socio-demographic variables including gender (codified as a dummy variable: 0 = male and 1 = female), age, employment status (codified as a dummy variable: 0 = unemployed and 1 = employed), and education (included using a simple effect coding with middle school diploma as reference group).

**Figure 1:**
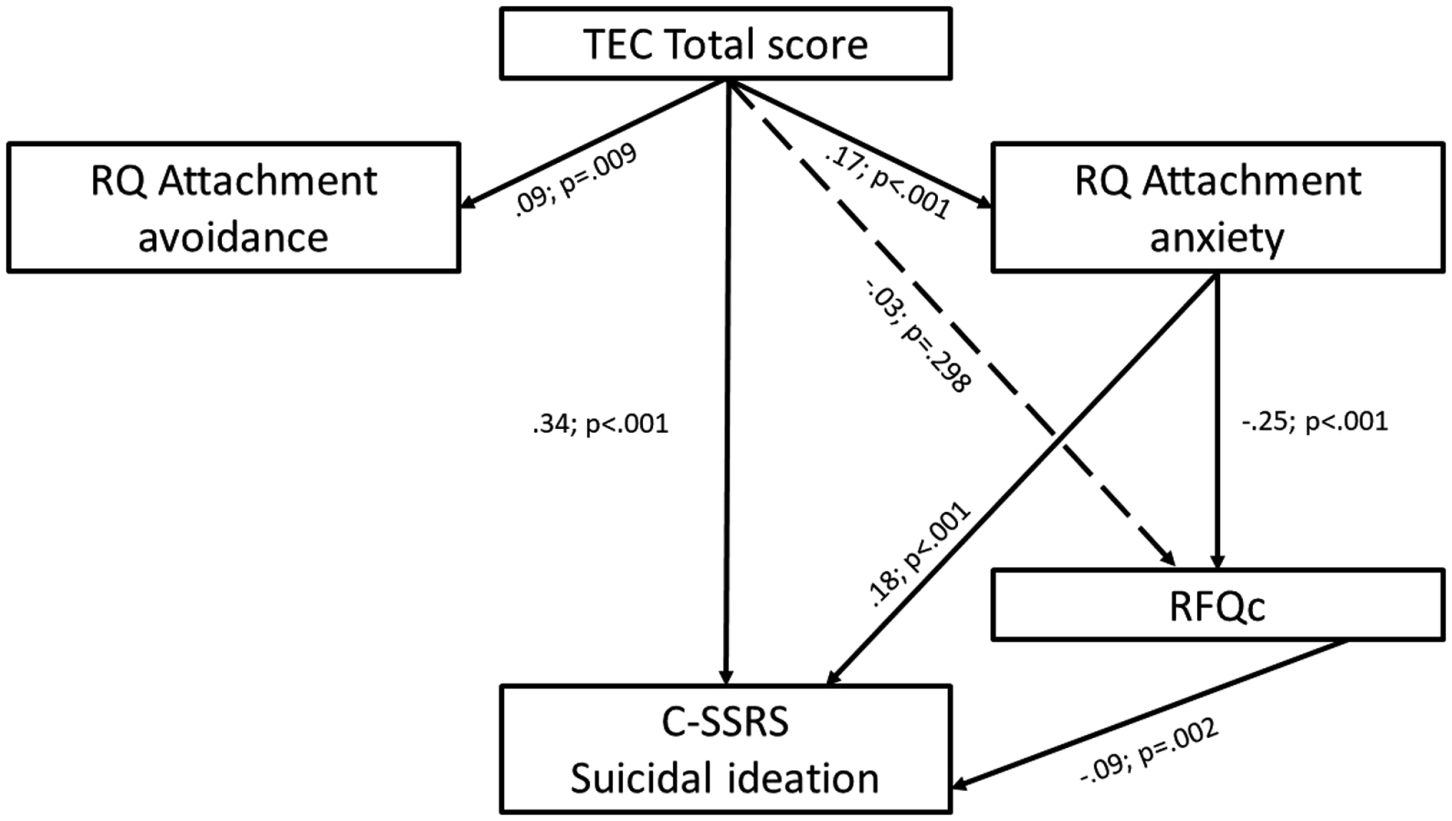
Path model of suicidal ideation as a function of the direct and indirect influences of traumatic experiences, being sequentially mediated by attachment anxiety and reflective functioning. Results are reported in completely standardized form. Dotted lines indicate non-significant effect at *p* > 0.05.

Afterwards, under the structural equation modeling framework, a path analysis with observed variables was conducted to test our mediation hypothesis (see Figure 1). In this case, we considered as the study outcome the summed score of the suicidal ideation subscale treated as a continuous variable in light of its six ordered categories (Rhemtulla et al., 2012). The observed variables did not present a significant departure from normality by analyzing their skewness and kurtosis values (Hair et al., 2010); hence, model parameters were estimated using the maximum likelihood (ML) estimator. The statistical significance of the indirect effects was assessed through the 95% bias-corrected bootstrap confidence intervals (BCI) calculated with 5,000 bootstrapping samples (Fritz and MacKinnon, 2007). Following a multifaceted conception of model fit (Tanaka, 1993), several indices were reported to assess the fit of the model to the data: Root Mean Square Error of Approximation (RMSEA; <0.05 indicates a close fit) (Browne and Cudeck, 1992), Standardized Root Mean Squared Residual (SRMR; <0.08 indicates a good fit) (Hu and Bentler, 1999), Tucker–Lewis index (TLI; >0.95 indicates a good fit) (Hu and Bentler, 1999) and Comparative Fit Index (CFI; >0.95 indicates a good fit) (Hu and Bentler, 1999). Chi square statistic was reported but not considered due to its overly sensitivity in case of large sample sizes (Hu and Bentler, 1999).

## Results

### Descriptive statistics and preliminary analyses

The sample consisted of 950 participants with a mean age of 27.12 (SD = ±8.94), ranging from 18 to 73. Nearly 85% of the participants were female (*n* = 819). Most of the respondents had a high school diploma (*n* = 411; 43.26%) and were employed (*n* = 190; 20%). Table 1 shows the socio-demographic characteristics of the participants.

**Table 1:** Socio-demographic characteristics of the participants.

	Mean	SD	Minimum	Maximum
Age	27.12	±8.94	18	73
	*n*		%	
**Gender**				
Female	819		86.21	
Male	129		13.58	
Other	2		0.21	
**Educational level**				
Secondary lower education	24		2.52	
Secondary upper education	411		43.26	
Bachelor’s degree	277		29.16	
Master’s degree	180		18.95	
PhD or postgraduate education	58		6.11	
**Marital status**				
Involved in a loving relationship	388		40.84	
Cohabitant/Married	210		22.11	
Separated/Divorced	16		1.68	
Widowed	2		0.21	
Single	334		35.16	
**Employment status**				
Student	407		42.84	
Working student	221		23.26	
Employee	190		20	
Freelancer	75		7.89	
Unemployed	57		6	

Moreover, 40.32% of the sample did not ever have any history of suicidal ideation. The most frequent attachment style (RQ) was fearful (35.89%), whereas the least frequent was preoccupied (17.79%). The most frequent traumatic experience was emotional neglect (7.68%) followed by emotional abuse (3.79%). Table 2 shows descriptive statistics of the variables under study. All the continuous variables reported in Table 2 showed skewness and kurtosis values below |2|, suggesting non-severe departures from univariate normality (e.g., George and Mallery, 2010; Hair et al., 2010).

**Table 2:** Descriptive statistics of the variables under study.

C-SSRS history of suicidal ideation	Frequency dichotomous outcome	No suicidal ideation		Suicidal ideation		
		383 (40.32%)		567 (59.68%)		
RFQ	Total score RFQc	**Range**	**Mean**	**SD**	**Median**	**Mode**
		0;18	5.63	±4.45	5	0
	Total score RFQu	**Range**	**Mean**	**SD**	**Median**	**Mode**
		0;18	4.84	±3.58	4	4
RQ	Total score	**Range**	**Mean**	**SD**	**Median**	**Mode**
	Attachment anxiety	−12;11	0.10	±4.64	0	1
	Attachment avoidance	−11;12	1.09	±4.31	1	2
	Frequency of attachment styles	**Secure attachment style**	**Fearful attachment style**	**Preoccupied attachment style**		**Dismissing attachment style**
		240 (25.63%)	341 (35.89%)	169 (17.79%)		200 (21.05%)
TEC	Total Score	**Range**	**Mean**	**SD**	**Median**	**Mode**
		0;20	4.53	±3.34	4	3
	Frequencies of scores	**0**	**1**	**2**		**3**
	Emotional neglect	358 (37.68%)	326 (34.32%)	193 (20.32%)		73 (7.68%)
	Emotional abuse	360 (37.89%)	419 (44.11%)	135 (14.21%)		36 (3.79%)
	Physical abuse	769 (80.85%)	154 (16.21%)	24 (2.53%)		3 (0.32%)
	Threat to life, pain, bizarre punishment	592 (62.32%)	313 (32.95%)	40 (4.21%)		5 (0.53%)
	Sexual harassment	710 (74.74%)	225 (23.68%)	14 (1.47%)		1 (0.11)
	Sexual abuse	824 (86.74%)	117 (12.32%)	5 (0.53%)		4 (0.42%)

Furthermore, preliminary analyses evaluating mean differences across history of suicidal ideation categories (i.e., presence vs. absence) on the continuous variables of interest, as well as the relationships between the suicidal ideation subgroups and the categorical variables under investigation, are reported in Supplementary Tables 1,2, respectively. Lastly, Pearson’s correlation coefficients between the suicidal ideation summed scores and the continuous variables under study are reported in Supplementary Table 3.

### Logistic regression model

Multiple logistic regression was employed by setting a history of suicidal ideation (i.e., presence vs. absence of suicidal ideation) as the model outcome, evaluating the predictive role of the RFQc, RFQu, attachment anxiety, and attachment avoidance dimensions, as well as of socio-demographic variables including gender (0 = male and 1 = female), age, employment status (0 = unemployed and 1 = employed), and education (included using a simple effect coding with middle school diploma as reference group) (see Table 3). Continuous variables were mean-centered before regression analysis with the aim of obtaining a meaningful intercept. Findings showed that the odd of reporting suicidal ideation was positively associated with attachment anxiety (OR = 1.09; *p* < 0.001) and TEC scores (OR = 1.23; *p* < 0.001), whilst was negatively related to RFQc scores (OR = 0.95; *p* = 0.007) and age (OR = 0.97; *p* < 0.001).

**Table 3:** Multiple logistic regression analysis with history of suicidal ideation as model outcome.

	Estimate	Standard error	Odds ratio	*z*	*p*	95% CI
(Intercept)	0.56	0.28	1.75	2.01	0.04	1.01 to 3.01
Age	−0.03	0.01	0.97	−3.33	<0.001	0.95 to 0.99
Gender	−0.07	0.21	0.94	−0.30	0.76	0.62 to 1.42
Secondary upper education	0.35	0.48	1.43	0.73	0.46	0.55 to 3.73
Bachelor’s degree	0.28	0.49	1.32	0.57	0.57	0.51 to 3.50
Master’s degree	0.49	0.50	1.63	0.99	0.32	0.62 to 4.39
PhD or Specialization school	0.78	0.55	2.19	1.42	0.16	0.74 to 6.58
Employment status	−0.03	0.16	0.97	−0.20	0.84	0.70 to 1.34
RFQc	−0.05	0.02	0.95	−2.70	0.007	0.92 to 0.99
RFQu	0.02	0.03	1.02	0.79	0.43	0.97 to 1.07
Attachment anxiety	0.09	0.02	1.10	5.09	<0.001	1.05 to 1.13
Attachment avoidance	0.02	0.02	1.02	1.27	0.20	0.99 to 1.06
TEC Total score	0.20	0.03	1.23	7.53	<0.001	1.16 to 1.29

Gender was codified as a dummy variable: 0 = male and 1 = female; Employment status was codified as a dummy variable: 0 = unemployed and 1 = employed; Education was included using a simple effect coding with middle school diploma as reference group.

### Path analysis

The path model depicted in Figure 1 demonstrated a satisfactory fit to the data: *χ*^2^(3) = 5.751, *p* > 0.05; RMSEA = 0.031 (95% CI 0.000 to 0.069; test of close fit *p* > 0.05); CFI = 0.991; TLI = 0.969; SRMR = 0.021. Findings showed that TLE were positively related to attachment avoidance (*β* = 0.09; *p* = 0.009). Also, TLE were positively associated with attachment anxiety (*β* = 0.17; *p* < 0.001), which in turn was negatively associated with RFQc (*β* = −0.25; *p* < 0.001) and positively associated with suicidal ideation (*β* = 0.18; *p* < 0.001). Still, RFQc negatively impacted on suicidal ideation (*β* = −0.09; *p* = 0.002).

The analysis of the indirect effects revealed two significant mediation paths. That is, the path between TLE and suicidal ideation *via* attachment anxiety was significant (*β* = 0.03; 95% BCI 0.02–0.05), as well as the sequential mediation of attachment anxiety and RFQc between TLE and suicidal ideation (*β* = 0.004; 95% BCI 0.001–0.008). Since a direct effect of TLE on suicidal ideation was found (*β* = 0.34; *p* < 0.001), a partial mediation model adequately fitted the observed data.

## Discussion

This study aimed at advancing the understanding of the link between TLE and suicidal ideation. Our findings advance the existing literature because they provide evidence for the sequential mediating roles of attachment anxiety and RF in this relationship.

As we expected (H1), our results confirmed the well-established positive association between TLE and suicidal ideation (Krysinska and Lester, 2010; Sorsdahl et al., 2011; Ásgeirsdóttir et al., 2018). Specifically, the results of the multivariate logistic regression analysis showed that individuals with a history of traumatic experiences had the highest odd ratio among the evaluated risk factors for suicidal ideation. This finding is consistent with the literature that highlighted the deleterious impact of cumulative exposure to traumatic experiences on mental health (Schimmenti, 2018).

In line with previous studies (Muller et al., 2000; Cozzarelli et al., 2003), our results also showed positive associations between TLE and both attachment anxiety and attachment avoidance. This finding is consistent with (Bowlby’s 1988) idea that attachment is potentially modifiable by disconfirming experiences of life events. In addition, attachment anxiety was also positively associated with suicidal ideation, thus partially mediating the relationship between TLE and suicidal ideation (H2). This is in line with previous studies that found associations between insecure attachment and increased risk for suicide (Salzinger et al., 2007; Grunebaum et al., 2010; Palitsky et al., 2013; Miniati et al., 2017; Wrath and Adams, 2019; Zortea et al., 2021; Danner Touati et al., 2022; Ihme et al., 2022). Consistent with and extending Adam’s model of suicide risk (1994), the lifetime suicidal ideation among adults with TLE has been found to be related with the development of internal working models characterized by fear of separation and fear of rejection. In fact, in times of distress, individuals with attachment anxiety appear to be hypersensitive to adverse experiences (Declercq and Willemsen, 2006) and adopt maladaptive coping strategies to assuage their unmet emotional needs (e.g., needs for acceptance, stability and bonding). This, in turn, may trigger an “attachment crisis,” resulting in suicidal ideation (Adam, 1994).

Also, we found that an aspect of RF (i.e., certainty about mental states) was negatively associated with suicidal ideation, thus supporting that the RFQc subscale of the RFQ captures individuals’ adaptive characteristics associated with better mental health (Garon-Bissonnette et al., 2022). In addition, the path analysis showed a sequential mediation effect of attachment anxiety and certainty about mental states between TLE and suicidal ideation (H3). This finding is in contrast with earlier work by Green et al. (2021) which did not find this mediation effect. These differences could be at least in part due to sample size (relatively small versus relatively large), characteristics (clinical versus community sample), and assessment (current versus lifetime suicidal ideation). However, our results are consistent with previous studies that showed that both insecure attachment and RF failures are positively associated with suicide risk (Stagaki et al., 2022). Although further studies are needed, our findings suggest that some individuals at risk of suicidal ideation had a history of TLE, high attachment anxiety, and difficulties in mentalization. One possible reason for this finding is that individuals who cannot engage in mentalization may have difficulties in understanding the source of their negative emotions, thus exacerbating reactions to traumatic memories (Doba et al., 2022). In these cases, anxious working models may interfere with an adequate mentalization of negative affect related to traumatic memories (e.g., by centering on feeling of helplessness) (Musetti et al., 2020, 2021).

In agreement with previous findings (e.g., Dennis et al., 2007), we found that younger adults reported higher rates of suicidal ideation than older adults. Moreover, as revealed by the logistic regression analysis, our results did not show significant gender differences in suicidal ideation. This finding is not consistent with the so-called “gender paradox” in suicide (Canetto and Sakinofsky, 1998), suggesting a higher risk for suicide among men and a higher risk for suicidal ideation among women. However, this result should be taken with caution due to the unbalanced sample sizes between gender groups.

The current study comes with limitations that should be carefully addressed. We found a life-time rate of suicidal ideation of 59.68%, which is quite higher than in other studies (Nock et al., 2008; Castillejos et al., 2021). This may be related to the characteristics of our sample which consisted mainly of university students, thus reflecting the heterogeneity of prevalence of suicidal ideation within this population (e.g., Coentre and Góis, 2018). Additionally, our sample was composed prevalently by females (86.21%). Although this may reflect the generally higher propensity of women to participate in online surveys (Kwak and Radler, 2002), we cannot exclude that snowball sampling may have led to bias in the selection of participants. Because of the observational cross-sectional design of this study, causal inferences cannot be drawn from the reported results. Importantly, in this study, we used the term “mediation” only in the statistical sense. Longitudinal studies are needed to disentangle the role played by RF in the relationship between attachment anxiety and suicidality. Although we used well-validated questionnaires, self-report instruments might have led to bias (e.g., recall bias); accordingly, future research should employ multimethod assessment (e.g., combining self-reported measures with structured or semi-structured clinical interviews) that provide more reliable information. Furthermore, as mentioned above, RF is supposed to include both trait and state features, therefore, an assessment limited to a single time point may not capture the fluctuations to which RF is subjected during events perceived as traumatic or highly stressful. Future research should also take into account how the age of exposure to trauma and the specific types of trauma influence adult attachment, RF, and ultimately suicidal ideation. Finally, we did not investigate if participants have ever had a history of psychiatric hospitalization or if they ever contacted the local mental health services for psychiatric or psychological support during or before the time of assessment. Therefore, we cannot speculate about the role of psychopathological symptoms in the investigated associations between variables.

### Implications for practice

Psychotherapeutic treatments have already been reported to be effective in reducing the rate of suicidal attempts in adults and adolescents, in comparison with patients allocated to receive usual treatment (Calati and Courtet, 2016). Our study supports the importance of assessing RF as part of psychosocial treatments, such as mentalization-based therapy (Luyten et al., 2020), to improve RF and emotional regulation capacity of patients, especially those with TLE. Clients with a history of TLE may need to experience a “secure base” in the treatment milieu to deal with painful feelings of unworthiness and rejection associated with anxious internal working models of attachment and gain more trust toward their own and others’ minds. Furthermore, our findings suggest that assessing TLE, attachment, and RF could help clinicians better understand the “functional significance” of suicidal ideation (see Lessard and Moretti, 1998). Lastly, prevention programs could be aimed at individuals with a history of traumas in order to foster their secure attachment and mentalization abilities.

## Conclusion

Notwithstanding its limitations, the present study shed new light on the role that TLE may play in the multidetermined processes underlying suicidal ideation. Specifically, that attachment anxiety and RF sequentially mediated the relationship between TLE and suicidal ideation. We conclude that difficulties in mentalization and attachment anxiety may increase the risk for suicidal ideation in some individuals with TLE. Our findings suggest that mentalizing abilities could reduce the vulnerability for suicidal ideation associated with feelings of unworthiness, inadequacy, and rejection that are rooted in attachment insecurities and traumatic memories.

## Data availability statement

The raw data supporting the conclusions of this article will be made available by the authors, without undue reservation.

## Ethics statement

The authors assert that all procedures contributing to this work comply with the ethical standards of the relevant national and institutional committees on human experimentation and with the Helsinki Declaration of 1975, as revised in 2008. The patients/participants provided their written informed consent to participate in this study.

## Author contributions

AM: conceptualization, investigation, project administration, writing–original draft, writing—review and editing, and supervision. LP and AZ: data curation, formal analysis, methodology, writing—original draft, and writing—review and editing. DU and SM: writing–original draft. VL and AG: writing—review and editing. CF and GG: writing—review and editing and supervision. All authors contributed to the article and approved the submitted version.

## Conflict of interest

The authors declare that the research was conducted in the absence of any commercial or financial relationships that could be construed as a potential conflict of interest.

## Publisher’s note

All claims expressed in this article are solely those of the authors and do not necessarily represent those of their affiliated organizations, or those of the publisher, the editors and the reviewers. Any product that may be evaluated in this article, or claim that may be made by its manufacturer, is not guaranteed or endorsed by the publisher.
