# Supplementary material for: Insecure adult attachment and reflective functioning as mechanisms of the relationship between traumatic life events and suicidal ideation: A path analysis
Source: Front Psychol. 2022 Sep 30;13:985148. doi: 10.3389/fpsyg.2022.985148 (PMC9561888; doi:10.3389/fpsyg.2022.985148)
Supplement: Supplementary file 1 [file Data_Sheet_1.docx]

**Supplementary Table 1.** Independent samples t-tests assessing mean differences across history of suicidal ideation categories (i.e., no suicidal ideation vs. suicidal ideation) on the continuous variables under investigation. Notes: ^a^Yuen’s t-test, also known as 20 percent trimmed means test, was implemented to compensate for non-normality and inequality of variances.

|  | | N | Mean | SD | t | df | p |
| --- | --- | --- | --- | --- | --- | --- | --- |
| Age | No suicidal ideation | 383 | 28.63 | ±10.33 | 2.975^a^ | 397.68^a^ | .003^a^ |
|  | Suicidal ideation | 567 | 26.10 | ±7.71 |  |  |  |
| Total score RFQc | No suicidal ideation | 383 | 6.57 | ±4.54 | 5.47 | 948 | <.001 |
|  | Suicidal ideation | 567 | 4.99 | ±4.28 |  |  |  |
| Total score RFQu | No suicidal ideation | 383 | 4.10 | ±3.06 | -5.31 | 948 | <.001 |
|  | Suicidal ideation | 567 | 5.34 | ±3.81 |  |  |  |
| Attachment anxiety | No suicidal ideation | 383 | -1.40 | ±4.33 | -8.54 | 948 | <.001 |
|  | Suicidal ideation | 567 | 1.12 | ±4.57 |  |  |  |
| Attachment avoidance | No suicidal ideation | 383 | 0.61 | ±4.22 | -2.88 | 948 | .004 |
|  | Suicidal ideation | 567 | 1.42 | ±4.35 |  |  |  |
| TEC Total Score | No suicidal ideation | 383 | 3.38 | ±2.56 | -9.10 | 948 | <.001 |
|  | Suicidal ideation | 567 | 5.31 | ±3.57 |  |  |  |

**Supplementary Table 2.** Chi-square tests of independence assessing relationships between history of suicidal ideation subgroups (i.e., no suicidal ideation vs. suicidal ideation) and the categorical variables under investigation.

|  | | | **C-SSRS Suicidal Ideation** | | **Χ^2^** | **df** | **p** |
| --- | --- | --- | --- | --- | --- | --- | --- |
|  |  |  | No suicidal ideation | Suicidal ideation |  |  |  |
| *Gender* | Male | N | 321 | 499 | 3.41 | 1 | 0.07 |
|  |  | % | 39.15% | 60.85% |  |  |  |
|  | Female | N | 62 | 68 |  |  |  |
|  |  | % | 47.69% | 52.31% |  |  |  |
| *Educational level* | Secondary lower education | N | 13 | 11 | 5.86 | 4 | 0.21 |
|  |  | % | 54.17% | 45.83% |  |  |  |
|  | Secondary upper education | N | 150 | 261 |  |  |  |
|  |  | % | 36.50% | 63.50% |  |  |  |
|  | Bachelor’s degree | N | 116 | 161 |  |  |  |
|  |  | % | 41.88% | 58.12% |  |  |  |
|  | Master’s degree | N | 78 | 102 |  |  |  |
|  |  | % | 43.33% | 56.67% |  |  |  |
|  | Ph. D or postgraduate education | N | 26 | 32 |  |  |  |
|  |  | % | 44.83% | 55.17% |  |  |  |
| *Marital*  *status* | Involved in a loving relationship | N | 150 | 238 | 15.67 | 5 | 0.003 |
|  |  | % | 38.66% | 61.34% |  |  |  |
|  | Cohabitant / Married | N | 102 | 108 |  |  |  |
|  |  | % | 48.57% | 51.43% |  |  |  |
|  | Separated / Divorced | N | 10 | 6 |  |  |  |
|  |  | % | 62.50% | 37.50% |  |  |  |
|  | Widowed | N | 2 | 0 |  |  |  |
|  |  | % | 100% | 0 |  |  |  |
|  | Single | N | 119 | 215 |  |  |  |
|  |  | % | 35.63% | 64.37% |  |  |  |
| *Employment status* | Unemployed | N | 172 | 292 | 3.97 | 1 | 0.046 |
|  |  | % | 37.07% | 62.93% |  |  |  |
|  | Employed | N | 211 | 275 |  |  |  |
|  |  | % | 43.42% | 56.84% |  |  |  |

**Supplementary Table 3.** Pearson’s correlation matrix. *p<.05, **p<.01, ***p<.001

|  | C-SSRS  Suicidal Ideation | Total score RFQc | Total score  RFQu | Attachment anxiety | Attachment  avoidance | TEC Total Score |
| --- | --- | --- | --- | --- | --- | --- |
| C-SSRS  Suicidal Ideation |  |  |  |  |  |  |
| Total score RFQc | -0.17*** |  |  |  |  |  |
| Total score RFQu | 0.18*** | -0.49*** |  |  |  |  |
| Attachment anxiety | 0.26*** | -0.25*** | 0.25*** |  |  |  |
| Attachment avoidance | 0.07* | -0.06 | 0.08* | 0.08* |  |  |
| TEC Total Score | 0.38*** | -0.07* | 0.18*** | 0.17*** | 0.09** |  |
